# Characterizing Variants of Unknown Significance of the PTEN tumour suppressorHomolog DAF-18

**DOI:** 10.17912/micropub.biology.000689

**Published:** 2022-11-28

**Authors:** Glafira Ermakova, Chadwick Hou, Jeffrey Boudreau, William G Bendena, Ian D Chin-Sang

**Affiliations:** 1 Queen’s University, Department of Biology, Kingston ON Canada

## Abstract

Insulin and insulin-like growth factor signaling (IIS) is an anabolic pathway conserved among humans and *Caenorhabditis elegans* . In humans, the tumour suppressor protein Phosphatase and Tensin Homolog (PTEN) inhibits IIS, preventing excessive growth. PTEN variants are associated with disease, but how they affect PTEN function is not well understood. Here, we characterized variants of unknown significance (VUSs) implicated in autism spectrum disorder by studying homologous mutations in the *C. elegans* protein DAF-18 to infer how they play a role in human disease.We found that variants D66E and L115V are likely benign, H168Q is intermediate while variants H138R and T176I are likely pathogenic.

**Figure 1. Variants of DAF-18 and their respective phenotypes.**
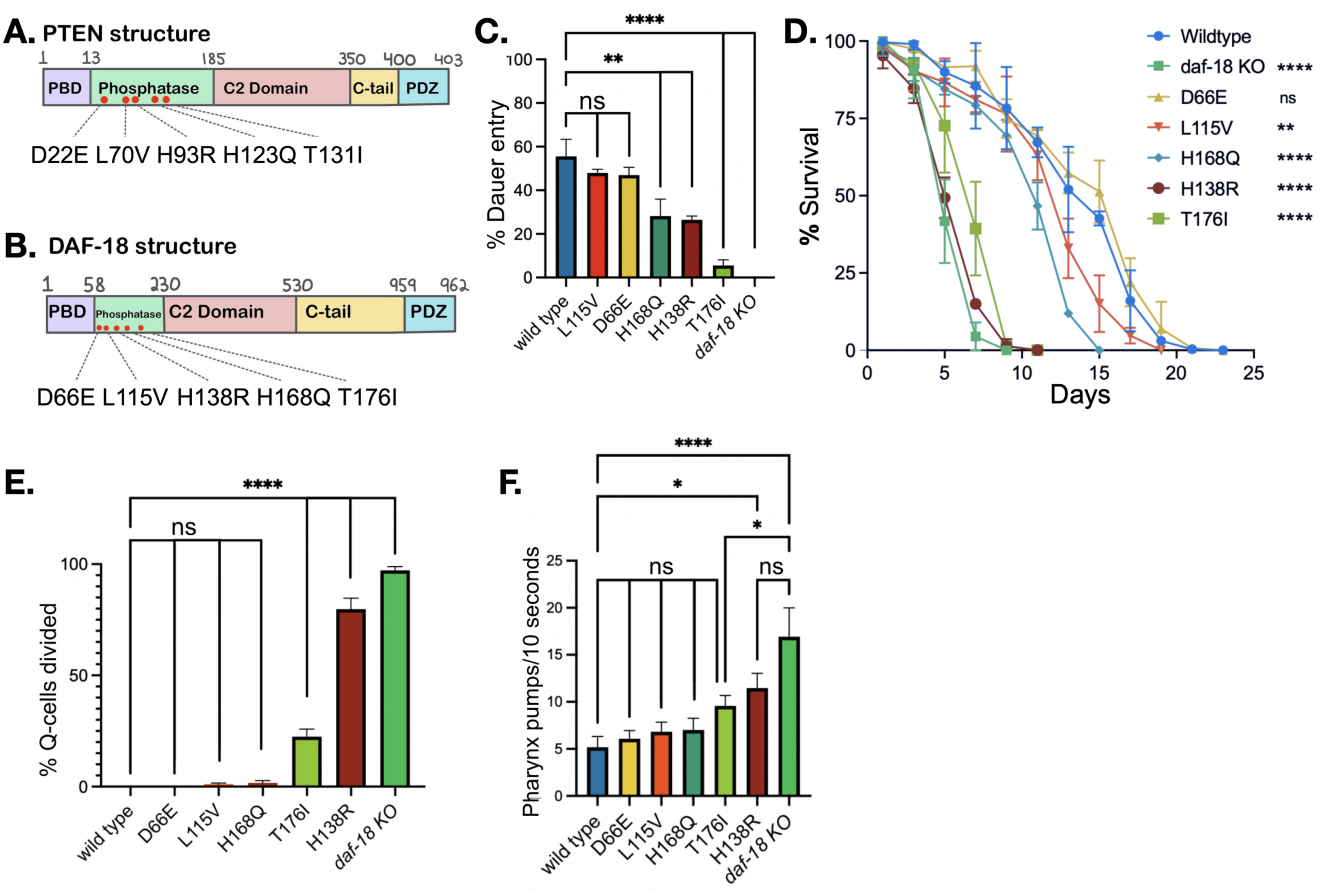
**A)** Structure of the human PTEN protein. Mutations D22E, L70V, H93R, H123Q, and T131I are illustrated with red. **B)** Structure of the *C. elegans* DAF-18 protein. Conserved DAF-18 mutations based on the PTEN variants, D66E, L115V, H138R, H168Q, and T176I in the phosphatase domain are illustrated with red. Adapted from Liu et. al. (2013), and Dirican & Akkiprik (2017). **C)** The graph shows the percentage of animals that enter dauer diapause after being exposed to dauer-inducing conditions (48 hours at 27 °C) in wild type, *daf-18(qu52)* knockout, and *daf-18* VUS strains. Graph shows mean + SEM of three independent experiments for dauer entry data, n = 100-150 per experiment. One-way ANOVA, Dunnett’s multiple comparisons test, **=p<0.009; ****=p<0.00009; ns = not significant. **D)** Survival of wild type, *daf-18(qu52)* knockout, and *daf-18* VUS strains in L1 arrest survival assay at 20 °C. n = 100-150 per strain at each day. Ordinary one-way ANOVA test was performed on the area under the curve value of each survival curve with at least three independent trials per strain. Error bars represent +/- SD of three independent trials at each timepoint (100-150 worms per day in each trial). **E)** The graph shows the average number of divided neuroblast Q-cells in wild type, *daf-18(qu52)* knockout, and *daf-18* VUS strain animals, recorded for day 5-8 in L1 arrest at 20 °C. Graph shows mean + SEM of three independent experiments for Q-cell division data, n = 100-150 per experiment. One-way ANOVA, Dunnett’s multiple comparisons test, ****=p<0.00009; ns = not significant. **F)** The graph shows average pharyngeal pumping activity of day 1 adult wild type, *daf-18(qu52)* knockout, and *daf-18* VUS strains recorded at 20 °C nematode growth medium. Pharyngeal pumping rate was measured as pumps per 10 seconds. Graph shows mean + SEM of three independent experiments for pharyngeal pumping data, n = 100-150 per experiment. One-way ANOVA, Dunnett’s multiple comparisons test, *=p < 0.05; ****=p<0.00009; ns = not significant.

## Description

Phosphatase and Tensin Homolog (PTEN) acts as a key suppressor of the insulin signaling pathway and thus regulates cell growth and development (Leslie et al., 2008). In humans, this protein still has numerous clinically important variants of unknown significance (VUS) which are implicated in disorders such as autism spectrum disorder (ASD) and are classified as neither pathogenic nor benign (Yehia et al., 2020). Our research aims to classify five of these variants previously implicated in ASD using the model organism *Caenorhabditis elegans* ( *C. elegans* ) (Wong et al., 2019). The insulin pathway is well conserved across metazoans, and thus using this model organism is advantageous as this allows us to characterize PTEN variants by studying its *C. elegans* homolog DAF-18 (Post et al., 2020). In 2019, Wong et al. characterized DAF-18 variants D66E, L115V, H138R, H168Q, and T176I, which correspond to PTEN variants D22E, L70V, H93R, H123Q, and T131I implicated in ASD (Figure 1A-B). Their analysis focused on morphology and locomotion defects, while González-Cavazos et al. in 2019 studied the effects of these variants on dauer entry. Our work aimed not only to replicate the results found by González-Cavazos et al., 2019 but also to characterize the DAF-18 variants with three new assays: L1 arrest survival, Q-cell division in L1 arrest, and rate of pharyngeal pumping off-food.

*C. elegans* enter dauer diapause under stressful conditions (such as crowding, starvation, and high temperature), which is typically accompanied by decreased insulin signaling (Karp, 2018). Measuring dauer entry is an effective way to indirectly assess the extent of activation of the insulin signaling pathway. In our work, we exposed *C. elegans* to high temperature (27 °C) for 48 hours, and dauer entry of wild type, *daf-18* knockout ( *qu52), daf-18(sy879)* [D66E], *daf-18(sy887)* [L115V], *daf-18(sy881)* [H138R], *daf-18(sy885)* [H168Q], and *daf-18(sy882)* [T176I] strain were observed. Results published by González-Cavazos et al., 2019 using dauer pheromone to induce dauer showed that variants *daf-18(sy879)* [D66E], *daf-18(sy887)* [L115V] and *daf-18(sy885)* [H168Q] were similar to wild type worms, whereas *daf-18(sy881)* [H138R] and *daf-18(sy882)* [T176I] variants were more dauer defective compared to wild type, with *daf-18(sy882)* [T176I] showing a stronger phenotype than *daf-18(sy881)* [H138R]. In our studies, we found that the *daf-18(sy879)* [D66E] and *daf-18(sy887)* [L115V] strains resembled wild type in terms of dauer entry, whereas *daf-18(sy885)* [H168Q], and *daf-18(sy882)* [T176I] were more dauer defective (Figure 1C). Overall, our findings mostly agreed with published data, with the exception of the *daf-18(sy885)* [H168Q] strain, though it did have more dauer entry than the dauer defective *daf-18(qu52)* control (Figure 1C).

Measuring L1 arrest survival is another way to assess activation of the insulin signaling pathway, with increased insulin signaling leading to shortened survival (Baugh et al., 2006; Chen et al., 2022). We thus assessed the L1 arrest survival ability of the VUS strains compared to wild type and *daf-18(qu52)* controls. Figure 1D shows the *daf-18(sy879)* [D66E] is not significantly different from wild type which further classifies this variant as most benign. The *daf-18* ( *sy887)* [L115V], and *daf-18(sy885)* [H168Q] live longer in L1 arrest than the *daf-18(qu52)* knockout and therefore are intermediate and not likely null alleles. The *daf-18(sy881)* [H138R] and *daf-18(sy882)* [T176I] worms have similar L1 arrest survival to that of the *daf-18(qu52)* knockout strain, with *daf-18(sy881)* [H138R] not being significantly different from the *daf-18(qu52)* knockout (One-way ANOVA, p=0.9706). This fits in with the published data and further characterizes D66E, L115V, and H168Q variants as benign and H138R and T176I as pathogenic (González-Cavazos et al., 2019). This assay reveals potential phenotype uncoupling with the *daf-18* (T176I) being more dauer defective than the *daf-18(sy881)* [H138R] strain, while having a higher survival in L1 arrest (One-way ANOVA p=0.0283).

Another readout of inappropriate activation of IIS in L1 arrest is the presence of divided, fluorescently tagged neuroblast precursor “Q-cells” (Zheng et al., 2018). We thus quantified Q cell division during L1 arrest of the VUS strains compared to wild type and *daf-18(qu52)* controls. Our experiments once again supported *daf-18(sy879)* [D66E], *daf-18(sy887)* [L115V], and *daf-18(sy885)* [H168Q] variants displaying a more benign phenotype while *daf-18(sy881)* [H138R] and *daf-18(sy882)* [T176I] variants were more pathogenic. Figure 1E shows that the *daf-18(sy881)* [H138R] and *daf-18(sy882)* [T176I] variants had some Q-cell division, which is indicative of an overactive IIS in L1 arrest and supports the deleterious effect of these variants. Notably, the phenotypes of *daf-18(sy881)* [H138R] and *daf-18(sy882)* [T176I] variants were not as strong as the *daf-18(qu52)* knockout control, which shows up to 100% Q-cell division in L1 arrest, suggesting that these variants are not completely null for *daf-18* function.

Pharyngeal pumping can be used to measure food intake, which is increased during IIS activation in *C. elegans* (Avery & You, 2018). Previous research shows that worms with an impaired IIS are unable to cease pumping while off food compared to wild type worms (Dallière et al., 2016). We thus quantified the pharyngeal pumping rate of the five DAF-18 VUS strains when taken off the food. We found that the *daf-18* knockout *C. elegans* had a higher pharyngeal pumping rate when taken off food (Figure 1F), as published. Of the VUS strains, *daf-18(sy879)* [D66E], *daf-18(sy887)* [L115V], *daf-18(sy885)* [H168Q], and *daf-18(sy882)* [T176I] were similar to wild type, whereas *daf-18(sy881)* [H138R] variant resembled the *daf-18* knockout (Figure 1F). This mostly supports our classification of *daf-18(sy879)* [D66E], *daf-18(sy887)* [L115V], and *daf-18(sy885)* [H168Q] as benign and *daf-18(sy881)* [H138R] and *daf-18(sy882)* [T176I] as potentially pathogenic, except for *daf-18(sy882)* [T176I], which could be due to phenotype uncoupling.

Together, the dauer entry, L1 arrest survival, Q-cell division in L1 arrest, and off-food pharyngeal pumping assays show that the D66E variant is most likely benign, being similar to wild type in all experiments. The L115V is also likely benign, being like wild type in 3 of the 4 assays. Meanwhile the H168Q variant appears to be intermediate in terms of its pathogenicity, showing phenotypic defects in half of the experiments. T176I is a stronger variant, with the *daf-18(sy882)* [T176I] allele conferring defects in all but the pharyngeal pumping assay. Meanwhile, the strongest allele appeared to be *daf-18(sy881)* [H138R], being significantly more deficient than wild type in all assays, and even being similar to the *daf-18(qu52)* knockout when it came to L1 arrest survival and pharyngeal pumping. Future work involving structural analysis of these variants could complement our findings. Furthermore, assessing VUSs of PTEN could be done without first needing to find and create homologous DAF-18 variants through replacement of *daf-18* with different variants of human *PTEN* via CRISPR. Our research thus helped characterize PTEN variants and has laid the groundwork for analyzing future clinically significant variants.

## Methods

***C. elegans* growth conditions:** *C. elegans* strains were grown on standard nematode growth medium agar plates (NGM) seeded with 100ml saturated culture *Escherichia coli (E. coli)* OP50 bacteria. Worms were kept in 20 °C at all times except during counting or unless stated otherwise.

**Dauer assay:** 100-150 synchronized L1 larvae were plated on seeded NGM plates and kept at 27 °C for 48 hours to induce dauer (for details, see Ailion & Thomas, 2000). SDS selection (1% SDS for 30 minutes) was then used to identify dauer and non-dauer worms, as non-dauer worms are killed by the SDS solution. The dauer *vs.* non-dauer (dead) animals were then manually counted. Three repeats of dauer *vs.* non-dauer count were done for each strain.

**L1 arrest survival assay:** *C. elegans* were placed in L1 arrest though sodium hypochlorite treatment and kept in 1.5ml tubes in M9 buffer on a rotator at 20 °C. M9 concentration was adjusted on day 1 of L1 arrest to obtain 10-15 worms per 1ml. Aliquots of 10ml were taken daily to count alive versus dead *C. elegans* (n=100-150 on each day). Three repeats were done for each strain. Statistics were carried out using GraphPad Prism software version 9.

**Q-cell division assay:** *C. elegans* were placed in L1 arrest using the methods described above. Q-cell division was scored manually throughout days 5-8 of L1 arrest by taking 10ml daily and counting the proportion of *C. elegans* with divided Q-cells (n=100-150 on each day). Division was visualized though a tagging the promoter of a protein expressed in the touch neurons ( *Pmec-4::GFP* ), and recording presence of the Q-cell descendant neuron, AVM (Zheng et al., 2018). Three repeats were done for each strain.

**Off-food pharyngeal pumping assay:** 100-150 synchronized day 1 adult *C. elegans* were washed three times with M9 buffer to remove bacteria. They were then plated onto NGM plates with no bacteria and pharyngeal pumping was manually measured (number of pumps per 10 seconds) after a 1-hour adjustment period.

## Reagents

*Strains:*

**Table:** 

Strain #	*Genotype*	Description
N2	*Wild type*	Wild isolate from Bristol
IC83	*zdIs5 [Pmec-4::GFP+lin-15(+)] I*	Touch neurons expressing GFP to visualize AVM and PVM (Q-cell descendants)
IC2384	*zdIs5 [Pmec-4::GFP+lin-15(+)]I;daf-18(sy879)IV*	Touch neuron marker with DAF-18 variant D66E
IC2385	*zdIs5 [Pmec-4::GFP+lin-15(+)]I;daf-18(sy887)IV*	Touch neuron marker with DAF-18 variant L115V
IC2386	*zdIs5 [Pmec-4::GFP+lin-15(+)]I;daf-18(sy885)IV*	Touch neuron marker with DAF-18 variant H168Q
IC2389	*zdIs5 [Pmec-4::GFP+lin-15(+)]I;daf-18(sy881)IV*	Touch neuron marker with DAF-18 variant H138R
IC2388	*zdIs5 [Pmec-4::GFP+lin-15(+)]I;daf-18(sy882)IV*	Touch neuron marker with DAF-18 variant T176I
IC2251	*zdIs5 [Pmec-4::GFP+lin-15(+)]I; daf-18(qu52) [GFP(+)::Δ daf-18] IV*	Touch neuron marker with daf-18 KO – allele *qu52* . The *daf-18(qu52)* allele is a Crispr/Cas9 edited strain where GFP is inserted at the start codon of *daf-18* locus, creating a transcriptional reporter and an out of frame *daf-18* knock out. Sequences are available upon request.
